# Supplementary material for: Interrogating the viral dark matter of the rumen ecosystem with a global virome database
Source: Nat Commun. 2023 Aug 29;14:5254. doi: 10.1038/s41467-023-41075-2 (PMC10465536; doi:10.1038/s41467-023-41075-2)
Supplement: Supplementary file 1 — Supplementary Information [file 41467_2023_41075_MOESM1_ESM.pdf]

Supplementary Information: Interrogating the viral dark matter of the rumen microbiome ecosystem with a global virome database

M. Yan et al.

Supplementary Figures

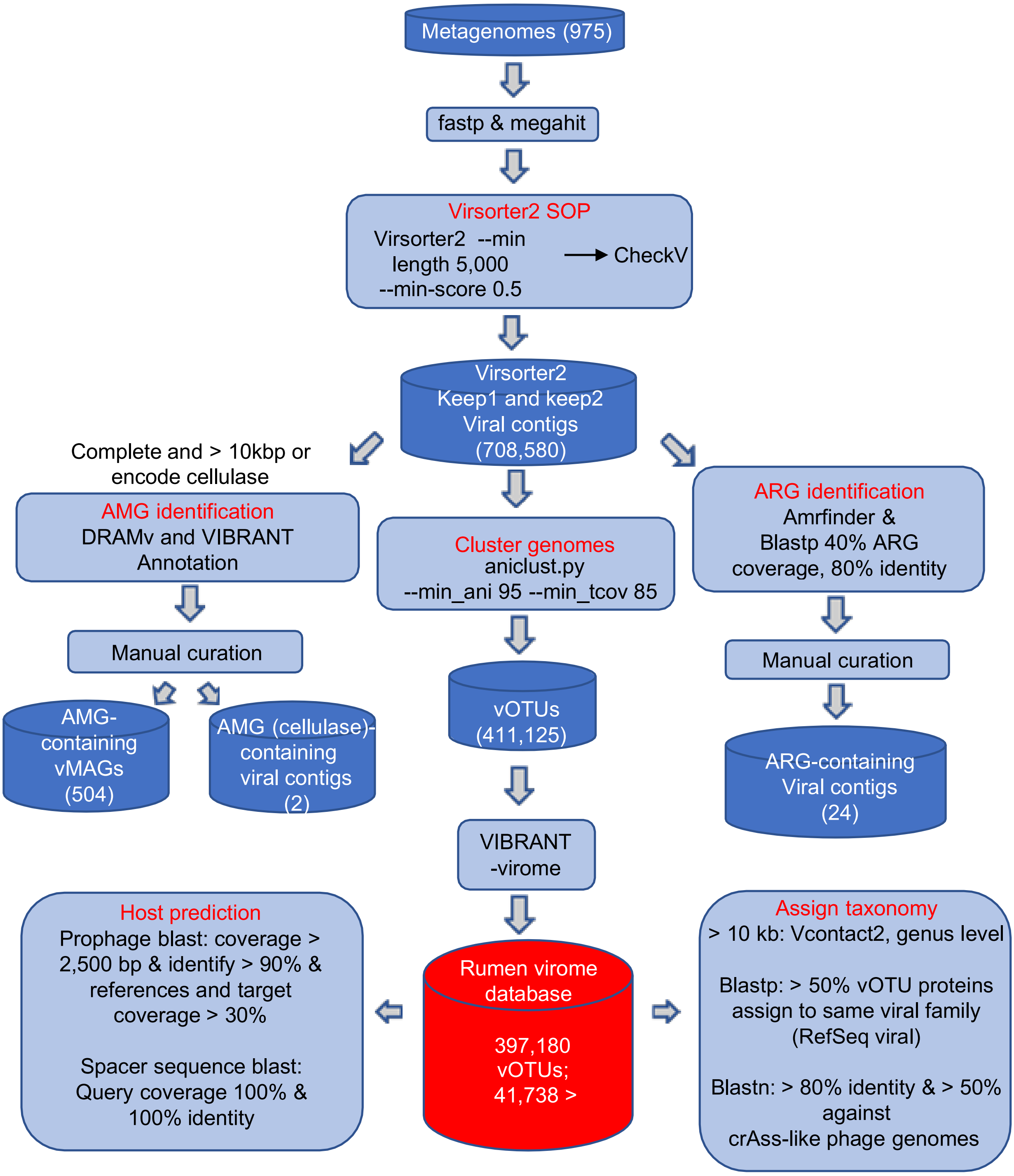

Supplementary Fig. 1: Workflow of the rumen virome analysis pipeline.

See also Supplementary Data 1 for detailed information about the metadata.

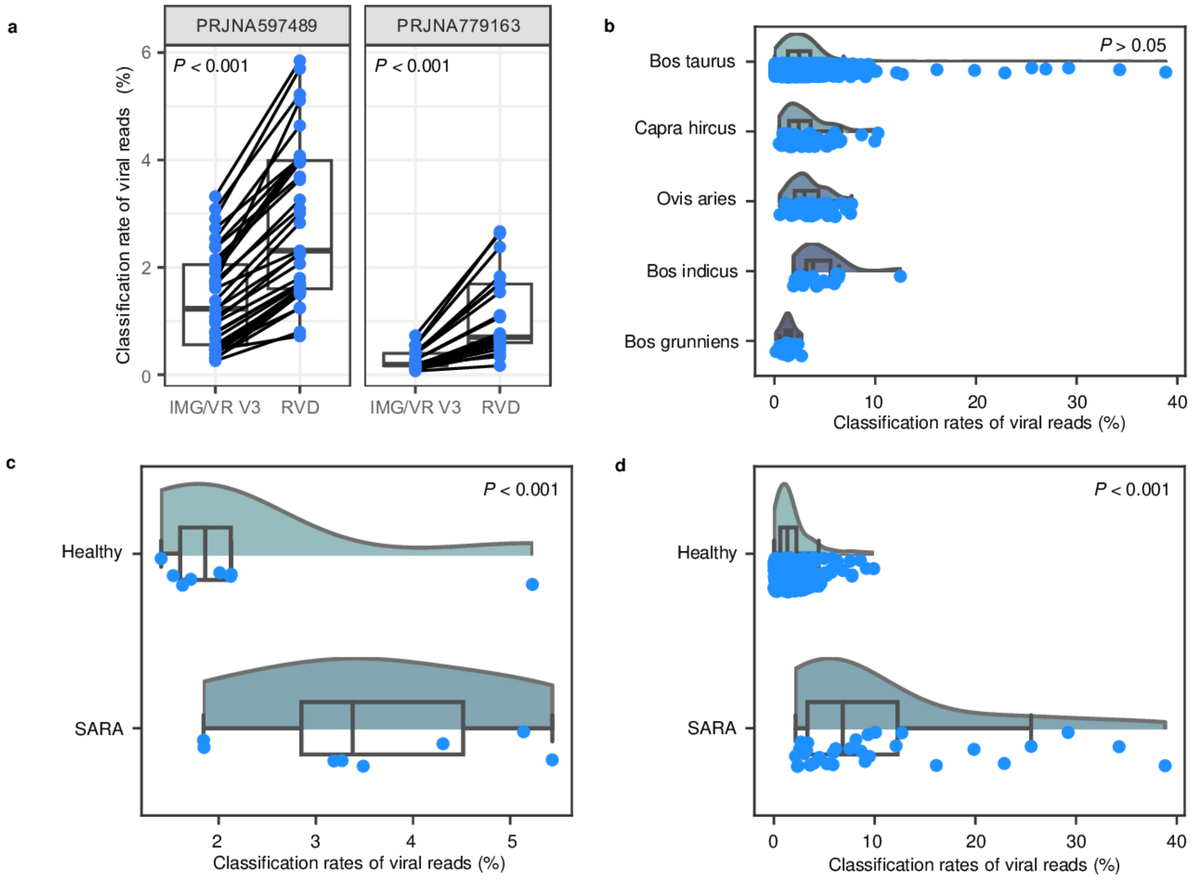

Supplementary Fig. 2: Mapping rates (%) of metagenomic sequence reads as viral sequences in metagenomes. a, Metagenomic sequencing reads across newly sequenced 33 rumen metagenomes from BioProject PRJNA597489 and 24 rumen metagenomes BioProject PRJNA779163 from with the host-associated fraction of IMG/VR V3[27] and RVD. **b,** Metagenomic sequencing reads from *Bos taurus* (n = 729), *Capra hircus* (n = 82), *Ovis aries* (n = 82), *Bos indicus* (n = 23), and *Bos grunniens* (n = 16). Ruminant species with less than 10 metagenomes were not included. **c,** Metagenomic sequencing reads from healthy goats (n = 8) and goats under subacute rumen acidosis (SARA; n = 8). Reads were obtained from NCBI (BioProject number: PRJNA552122). **d,** Metagenomic sequencing reads in healthy dairy cows (n = 203) and dairy under SARA (n = 48). Statistical significance in **a** - **d** was tested using the two-sided Wilcoxon signed-rank test. Box plots indicate the median (middle line), 25th, 75th percentile (box), and 5th and 95th percentile (whiskers) as well as individual observations (dots).

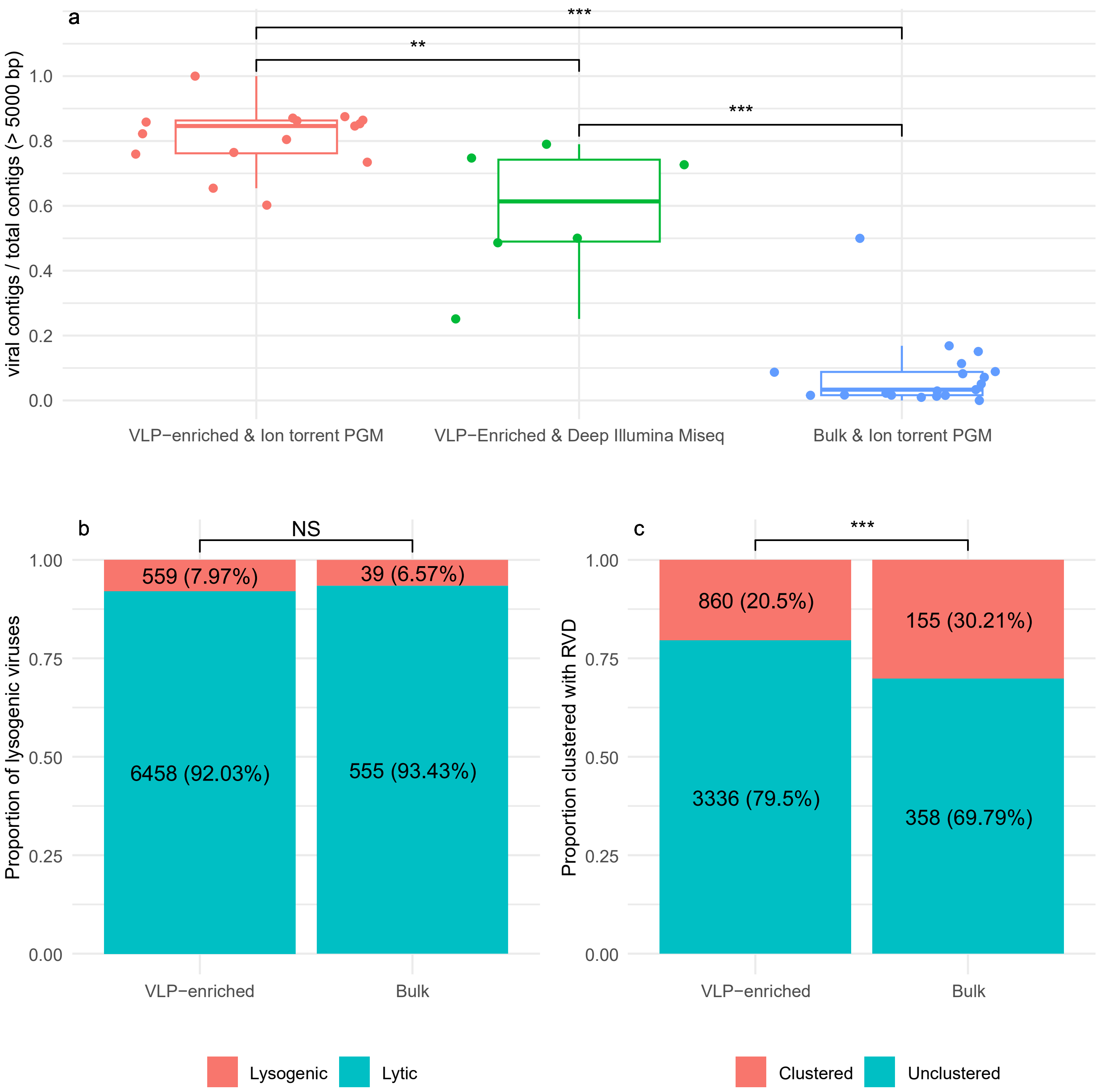

Supplementary Fig. 3: The effect of viral enrichment on viral identification.
a, The effect of viral enrichment on viral recovery rate (number of vial contigs/all contigs used for viral identification). **b,** Proportion of viral contigs of lysogenic viruses and viral contigs of lytic viruses recovered from bulk metagenomes and viral-like particle (VLP) enriched metagenomes. **c,** Proportion of vOTUs recovered from bulk metagenomes and VLP-enriched metagenomes represented in RVD. The values inside the bars in **b** and **c** designate the number of viral vOTUs and their percentage (between parenthesis), respectively. Statistical significance in **a** is tested with the pairwise Wilcoxon rank-sum test, while statistical significance in **b** and **c** is tested using the Chi-squared test. **, $P \leq 0.01$; ***, $P \leq 0.001$; NS, $P > 0.05$. Box plots indicate the median (middle line), 25th, 75th percentile (box), and 5th and 95th percentile (whiskers) as well as individual observations (dots).

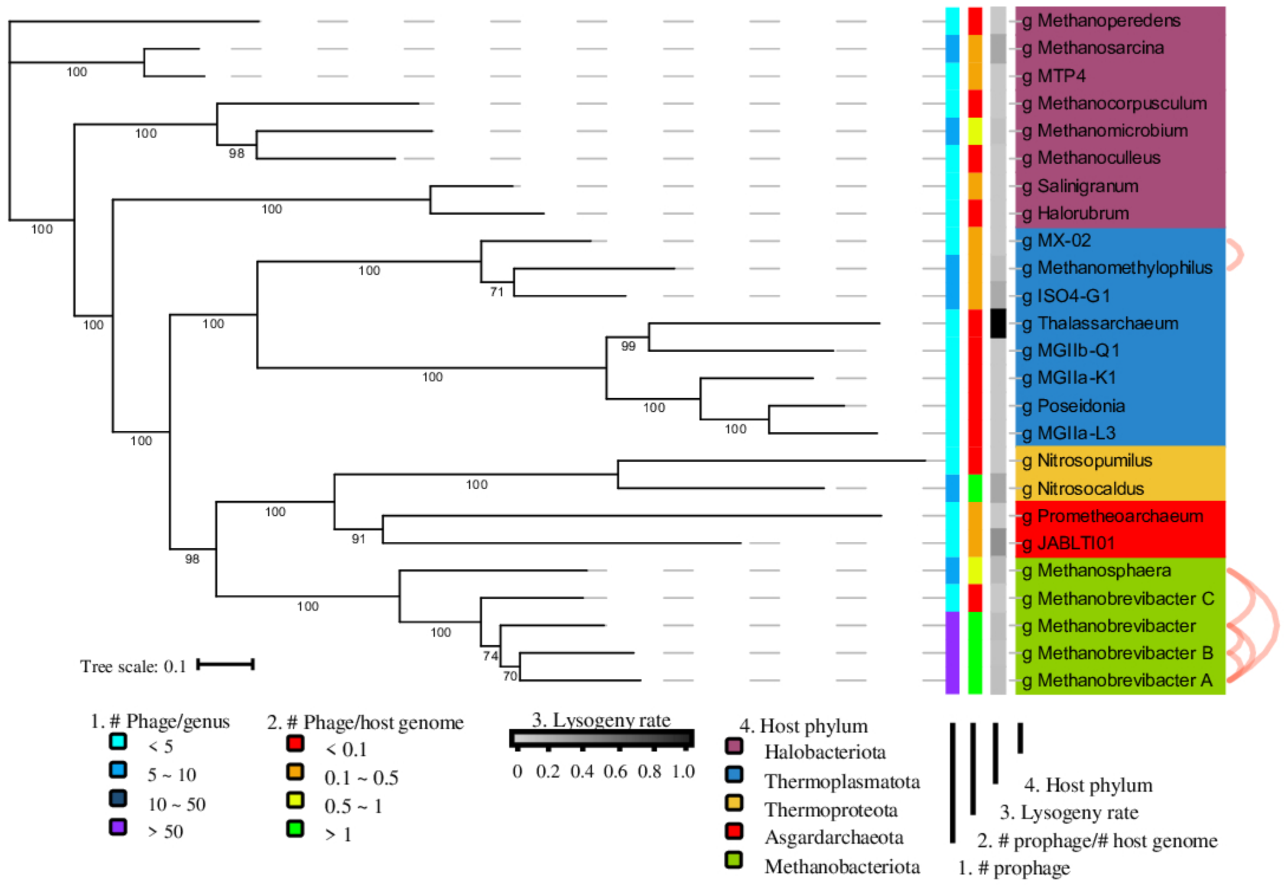

Supplementary Fig. 4: Predicted archaeal host of the rumen viruses.
For vOTU with host species across multiple genera, the predicted host genera were connected with orange arcs. The dendrogram represents the genome-based phylogenetic tree of 25 archaeal genera that contained the predicted hosts of 2,403 vOTUs. The hosts were inferred by (i) aligning the sequences of the representative vOTUs (the longest with the highest completeness) of each vOTUs with 410 metagenome-assembled genomes (MAGs) of rumen archaea and 8,367 bacterial reference genomes of NCBI RefSeq and (ii) aligning the CRISPR spacer sequences of the representative vOTUs and those of the RefSeq archaeal genomes and MAGs. The RefSeq archaeal genomes and MAGs were classified using GTDB-Tk. The phylogenetic tree was constructed with the genomes or MAGs of the inferred hosts (clustered into genera) and their predicted phages to examine the lysogeny rate (proportion, calculated based on the VIBRANT results), number of phages per host genus, and number of phages per host genome/MAG.

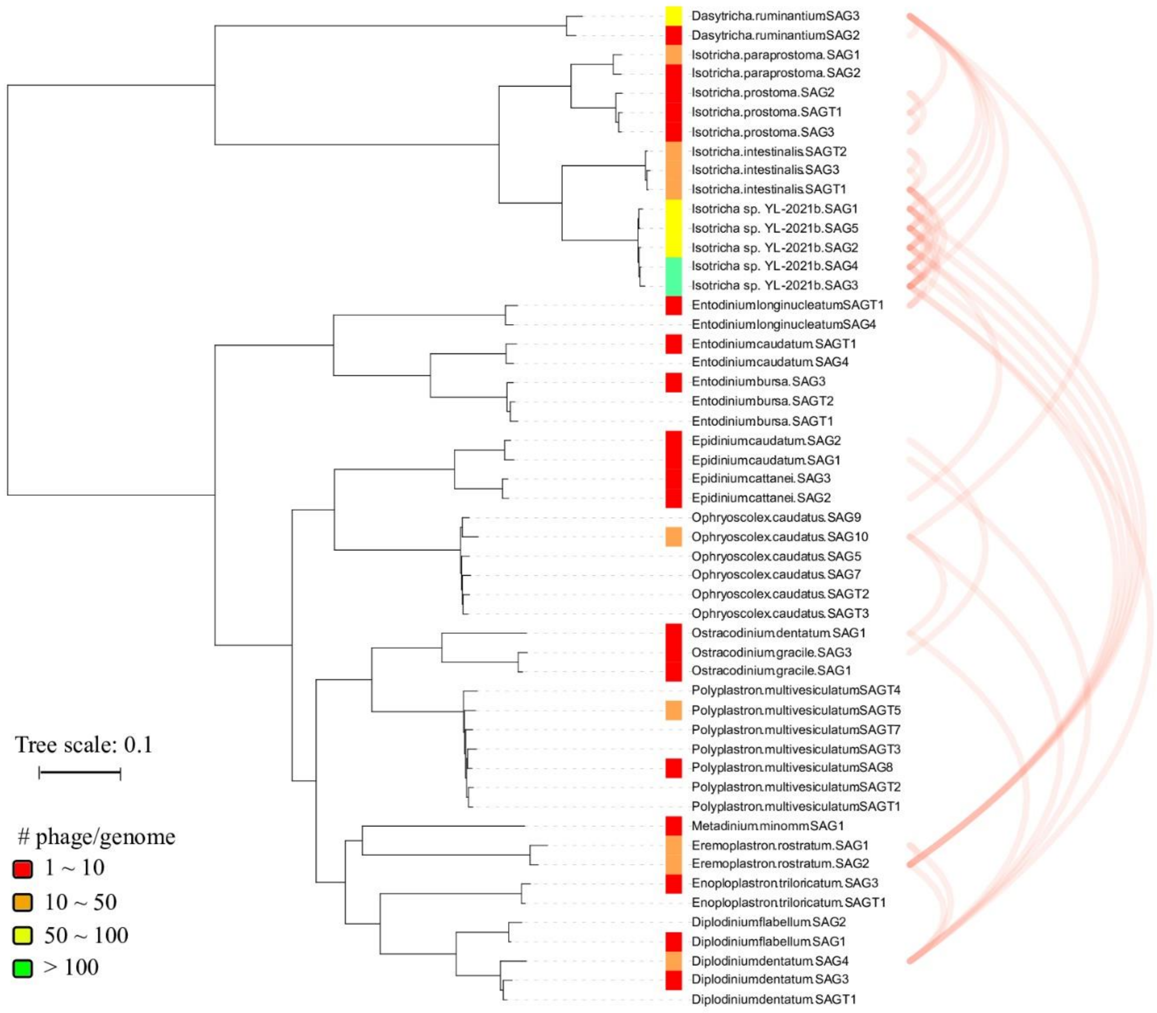

Supplementary Fig. 5: Predicted protozoal host of the rumen viruses.
The host genomes used for host prediction were the 52 recently reported high-quality single amplified genomes (SAGs)[68], and the dendrogram represents the phylogenetic tree of the 52 SAGs. In total, 500 vOTUs were predicted to affect rumen ciliates. For vOTUs with a host range across multiple genomes, the host genomes were connected with orange arcs. The phylogenetic tree was annotated according to the number of predicted prophages per SAG.

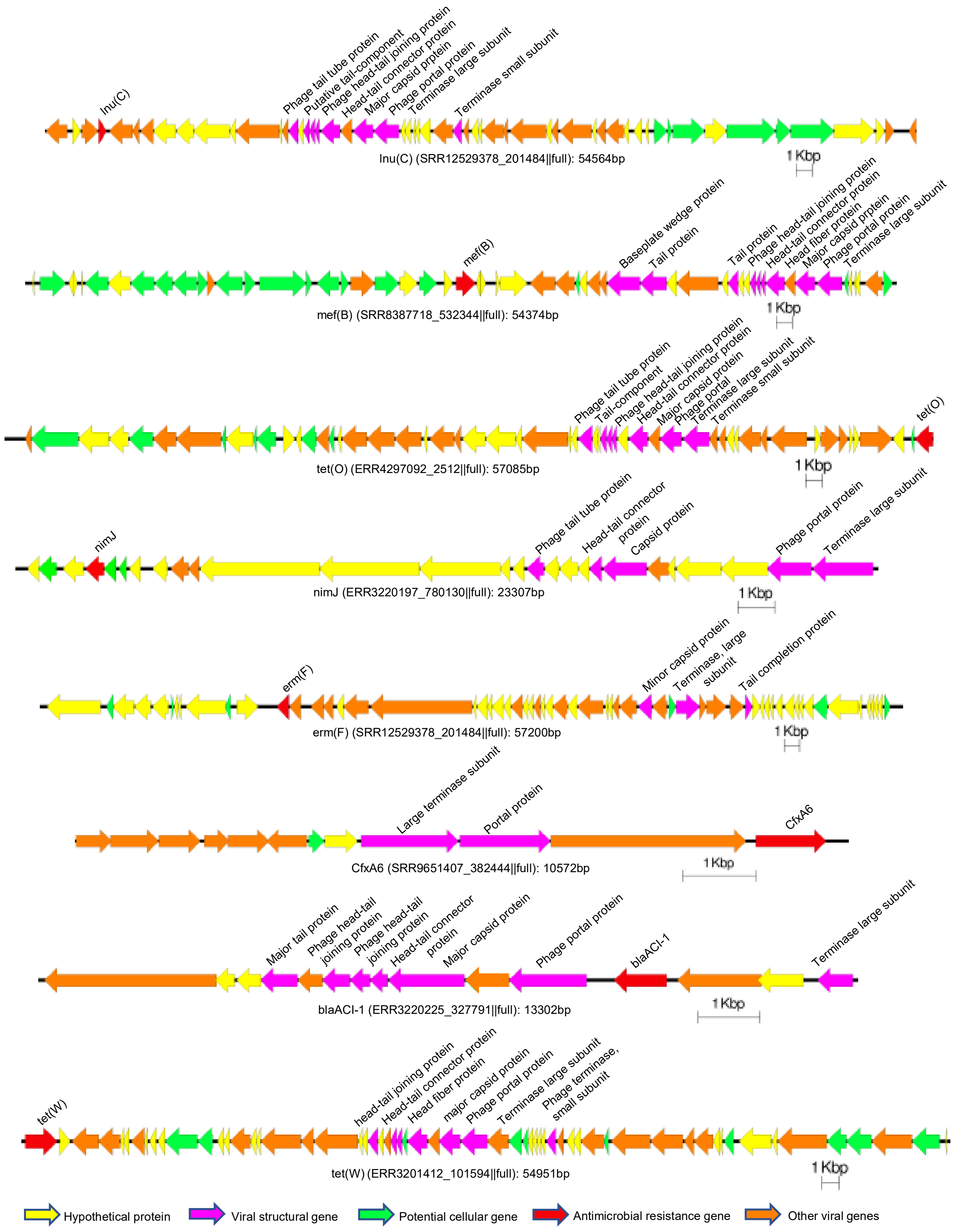

Supplementary Fig. 6: Genomic organization of representative viral contigs carrying each type of ARGs. Representative viral contigs were chosen from each vOTU that had the highest completeness and least host genes.

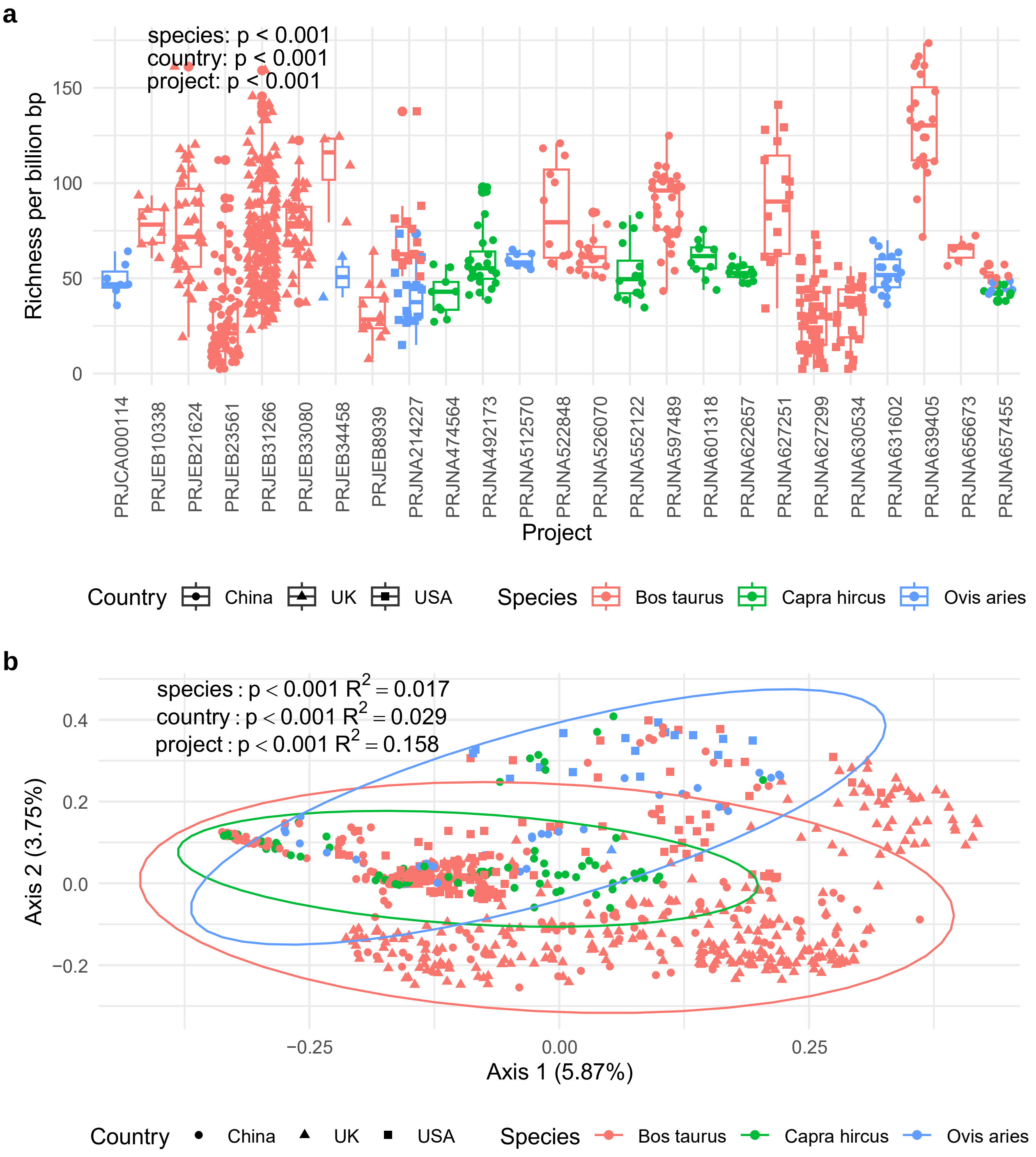

Supplementary Fig. 7: Ecological analysis of the rumen virome. a, Alpha-diversity (viral richness per billion bp of sequences as a proxy) results of samples from different ruminant species and countries. Only ruminant species or countries with more than 80 samples were included in the analysis. The Kruskal-Wallis test was used to compare the results among different ruminant species, countries, and projects. **b,** Beta-diversity results of samples from different ruminant species and countries. Permutational multivariate analysis of variance (PERMANOVA) was used to compare the overall virome in different ruminant species, countries, and projects. Statistical significance in **a** is tested with the Kruskal–Wallis test. Box plots indicate the median (middle line), 25th, 75th percentile (box), and 5th and 95th percentile (whiskers) as well as individual observations (dots).

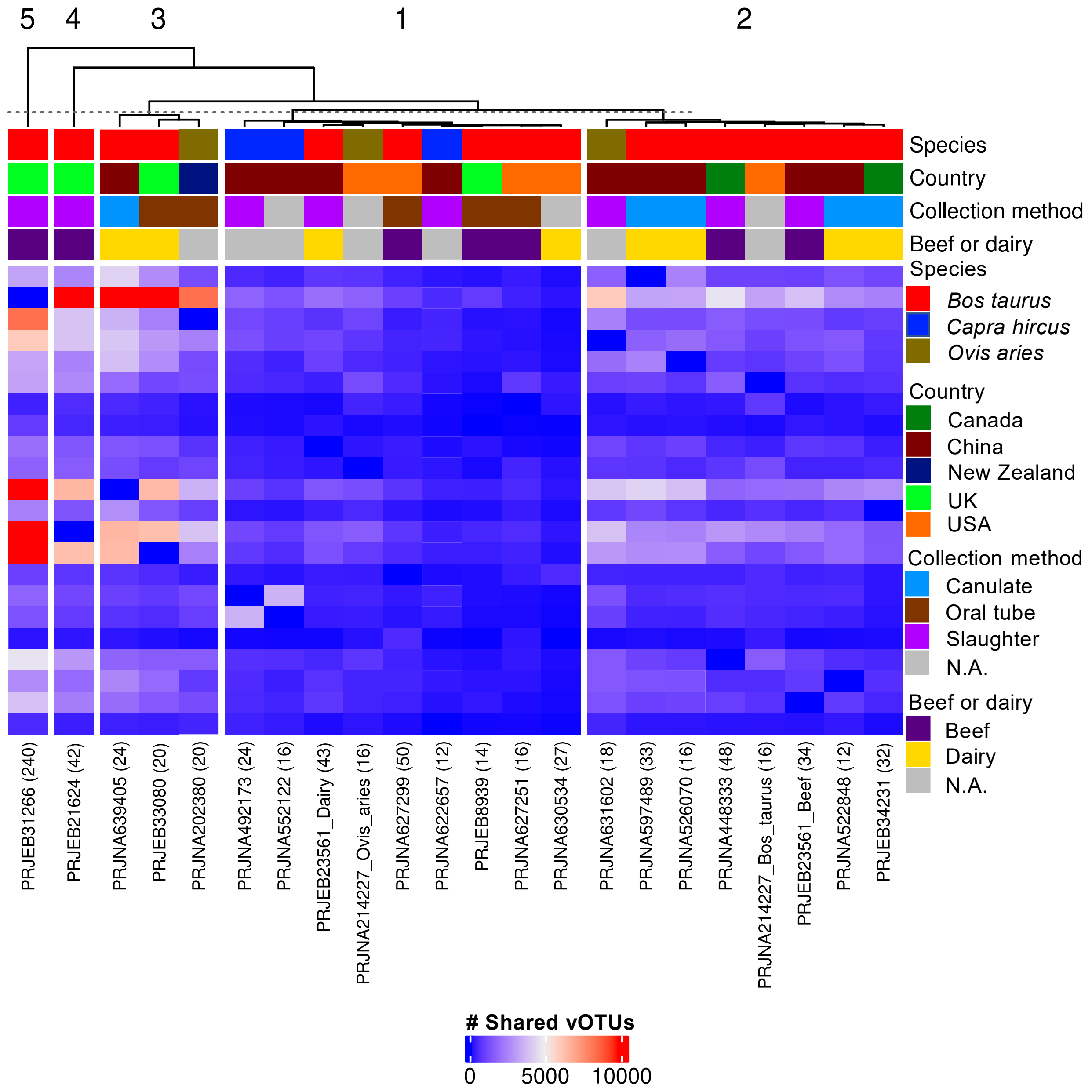

Supplementary Fig. 8: Cross-study comparisons of the rumen viral populations with a hierarchical clustering of the number of vOTUs shared between studies. N.A, information not available. The column names represent the project id and number of metagenomes (in parenthesis). The numbers (1 – 5) at the top indicate the clusters.
