# Supplementary material for: Interrogating the viral dark matter of the rumen ecosystem with a global virome database
Source: Nat Commun. 2023 Aug 29;14:5254. doi: 10.1038/s41467-023-41075-2 (PMC10465536; doi:10.1038/s41467-023-41075-2)
Supplement: Supplementary file 3 — Description of Additional Supplementary Files [file 41467_2023_41075_MOESM3_ESM.docx]

**Description of Additional Supplementary Files**

**File Name:** Supplementary Data 1

**Description:** The rumen metagenomes analysed in this study.

**File Name:** Supplementary Data 2

**Description:** Family- and genus-level assignment of the rumen viruses.

**File Name:** Supplementary Data 3

**Description:** Host prediction of the identified viruses of rumen prokaryotes.

**File Name:** Supplementary Data 4

**Description:** Curation of AMG-carrying vOTUs and AMG annotation.

**File Name:** Supplementary Data 5

**Description:** Antibiotic resistance genes carrying viral contigs annotations.

**File Name:** Supplementary Data 6

**Description:** Core rumen virome among cattle fed diet with different levels of concentrate and the probable hosts.
